# Producing Standardized Country-Level Immunization Delivery Unit Cost Estimates

**DOI:** 10.1007/s40273-020-00930-6

**Published:** 2020-06-28

**Authors:** Allison Portnoy, Kelsey Vaughan, Emma Clarke-Deelder, Christian Suharlim, Stephen C. Resch, Logan Brenzel, Nicolas A. Menzies

**Affiliations:** 1 Harvard T.H. Chan School of Public Health, Center for Health Decision Science, 718 Huntington Avenue 2nd Floor, Boston, MA 02115 USA; 2 Thinkwell, Washington, DC USA; 3 Department of Global Health and Population, Harvard T.H. Chan School of Public Health, Boston, MA USA; 4 Management Sciences for Health, Boston, MA USA; 5 Bill & Melinda Gates Foundation, Seattle, WA USA

## Abstract

**Background:**

To plan for the financial sustainability of immunization programs and make informed decisions to improve immunization coverage and equity, decision-makers need to know how much these programs cost beyond the cost of the vaccine. Non-vaccine delivery cost estimates can significantly influence the cost-effectiveness estimates used to allocate resources at the country level. However, many low- and middle-income countries (LMICs) do not have immunization delivery unit cost estimates available, or have estimates that are uncertain, unreliable, or old. We undertook a Bayesian evidence synthesis to generate country-level estimates of immunization delivery unit costs for LMICs.

**Methods:**

From a database of empirical immunization costing studies, we extracted estimates of the delivery cost per dose for routine childhood immunization services, excluding vaccine costs. A Bayesian meta-regression model was used to regress delivery cost per dose estimates, stratified by cost category, against a set of predictor variables including country-level [gross domestic product per capita, reported diphtheria-tetanus-pertussis third dose coverage (DTP3), population, and number of doses in the routine vaccination schedule] and study-level (study year, single antigen or programmatic cost per dose, and financial or economic cost) predictors. The fitted prediction model was used to generate standardized estimates of the routine immunization delivery cost per dose for each LMIC for 2009–2018. Alternative regression models were specified in sensitivity analyses.

**Results:**

We estimated the prediction model using the results from 29 individual studies, covering 24 countries. The predicted economic cost per dose for routine delivery of childhood vaccines (2018 US dollars), not including the price of the vaccine, was $1.87 (95% uncertainty interval $0.64–4.38) across all LMICs. By individual cost category, the programmatic economic cost per dose for routine delivery of childhood vaccines was $0.74 ($0.26–1.70) for labor, $0.26 ($0.08–0.67) for supply chain, $0.22 ($0.06–0.57) for capital, and $0.65 ($0.20–1.66) for other service delivery costs.

**Conclusions:**

Accurate immunization delivery costs are necessary for assessing the cost-effectiveness and strategic planning needs of immunization programs. The cost estimates from this analysis provide a broad indication of immunization delivery costs that may be useful when accurate local data are unavailable.

**Electronic supplementary material:**

The online version of this article (10.1007/s40273-020-00930-6) contains supplementary material, which is available to authorized users.

## Key Points for Decision Makers

**Table:** 

Immunization delivery costs are a necessary component of high-quality cost-effectiveness models, and are also used to inform resource mobilization for immunization programs.
Our study provides estimates produced via meta-regression analyses that can help improve resource mobilization and planning in situations where empirical cost data are unavailable or of low quality.

## Background

Routine immunization is critical to achieving 14 of the 17 Sustainable Development Goals (SDGs) adopted by countries in 2015 to “ensure prosperity for all” [1]. To monitor progress towards these goals, to plan for the financial sustainability of immunization programs, and to improve program coverage and equity, decision-makers need to know how much immunization programs cost beyond the cost of the vaccine. Non-vaccine immunization delivery unit cost estimates are essential for resource mobilization and planning for routine health systems, which have required the development of new financing mechanisms to support the increasing costs of country immunization programs [2]. Moreover, delivery cost estimates can help to identify and evaluate strategies to improve efficiency in vaccine service delivery [3]. Cost estimates can significantly influence the cost-effectiveness estimates used to allocate resources at the country level, particularly as service delivery can be the main driver of immunization program costs [4, 5]. In addition, low- and middle-income countries (LMICs) often require an updated immunization program costing to apply for support from Gavi, the Vaccine Alliance [3, 6]. Despite the multiple uses of accurate cost estimates, many LMICs have not conducted an empirical study of immunization services costs, or rely on estimates that are highly uncertain, unreliable, or old. While this evidence gap has been narrowed by recent efforts to improve the production and collation of immunization costing data—with 37 LMICs represented in available costing studies [7–9]—these efforts would need to be expanded substantially in order to supply all countries with up-to-date and high-quality cost estimates.

The objective of this study was to produce standardized country-level estimates of immunization delivery unit costs for all countries meeting the World Bank’s LMIC classification (136 total) [10], via an evidence synthesis of available data on immunization delivery costs [9, 11]. Using these data, we fit a Bayesian meta-regression model for routine childhood (i.e., under-five) vaccination program delivery unit costs. We present estimates of immunization delivery cost per dose by year, by cost category (labor, supply chain, capital, and other service delivery costs), and by country.

## Methods

We developed a Bayesian meta-regression model to predict immunization delivery unit costs, excluding vaccines and supplies such as syringes and safety boxes, in LMICs, according to World Bank country income classification in 2019 [10]. In this section, we describe the data used to fit the model, modeling methods, and alternative regression specifications.

### Data Extraction

We relied on a publicly available database describing immunization delivery costs in LMIC settings—the Immunization Delivery Cost Catalogue (IDCC) maintained by the Immunization Costing Action Network (ICAN) [9, 11]. The IDCC is an online web catalog and downloadable Excel spreadsheet of immunization delivery cost evidence in LMIC settings, which describes the results of a systematic review of published and grey literature available between January 2005 and March 2019. The search of the peer-reviewed literature included six major electronic databases, including EconLit, Embase, Medline (via PubMed), NHS Economic Evaluation Database (NHS-EED), Web of Science, and World Health Organization (WHO) Global Index Medicus. Search terms included three categories of keywords— “immunization” AND “cost” AND “delivery”—translated into the query language of each database. All resources with full-text availability in English, French, or Spanish, conducted in LMIC settings, that included a form of delivery unit cost data from primary data collection were included. In addition to extracting relevant contextual and methodological information from each study, the IDCC presents cost estimates in 2016 US dollars [9, 11].

From the IDCC, we identified studies that reported delivery cost per dose of routine (i.e., fixed facility) vaccine delivery, and which specified the cost categories included in the estimate. We excluded 39 studies that did not report a cost per dose or for which a cost per dose could not be calculated, studies that did not define the cost categories included in the cost per dose, and studies that focused solely on the costs of vaccines delivered through outreach or through supplementary immunization activities (SIAs), i.e., mass vaccination campaigns.

For the identified studies, we extracted estimates of the routine delivery cost per dose for childhood immunization, defined as vaccination of children under 5 years of age. We also extracted study-specific contextual information, including the number of sampled sites, when reported by a study, whether the study examined programmatic (i.e., full) cost per dose or single antigen (i.e., the incremental delivery costs of a single new vaccine) cost per dose, economic and/or financial cost per dose, and cost category cost per dose. An economic cost analysis estimates the annualized value of capital investments and the value of donated goods and labor time; whereas, a financial cost estimate is based on the financial outlay for capital equipment and excludes the value of donated goods and services [7]. If any information reported in the IDCC was unclear for this analysis, we confirmed the information in the original studies. For the observed cost per dose outcome, we extracted the observation with the highest level of granularity available from a given study. For example, if the total vaccine delivery cost per dose was also reported as individual cost categories (i.e., labor, supply chain, capital, and other service delivery), we extracted these data as independent observations and excluded the total vaccine delivery cost per dose. “Supply chain” includes costs for cold chain equipment, vehicles, transport, and fuel; “other service delivery” includes costs for program management (i.e., supervision and monitoring), training, social mobilization, and disease surveillance [6, 7].

We compiled data on covariates potentially associated with immunization delivery unit costs: the year of data collection (*Year*), a proxy for time-varying characteristics not captured by other model covariates; log number of doses in the routine vaccination schedule [log(*Doses*)], an indicator of the economies of scale for vaccine delivery; log gross domestic product (GDP) per capita [log(*GDP*)], an indicator of country price levels; reported diphtheria-tetanus-pertussis third dose coverage (*DTP3*), an indicator of routine health system capacity and overall coverage of the immunization program; and log total country population [log(*Pop*)], an indicator of service delivery volume [12, 13]. Model covariates log(*Doses*), log(*GDP*), and log(*Pop*) were compiled for the year of data collection for each study. By using a log transformation of specified covariates, we assumed these explanatory factors relate to the outcome on the multiplicative scale rather than linearly (for example, a doubling in per capita GDP produces a fixed increase in the outcome). The number of doses in the routine vaccination schedule was derived by reviewing country immunization schedules [14]. As historical vaccination schedules were not available for all calendar years, we assumed that the schedule for the most recently available year prior to the reported base year of the study applied.

### Prediction Model

We used a Bayesian meta-regression model to regress immunization delivery unit costs against country-level and study-level explanatory variables (Table 1). Continuous variables [*Year*, log(*Doses*), log(*GDP*), *DTP3*, and log(*Pop*)] were standardized to mean zero and unit standard deviation before fitting the regression model. We adopted an analytic model that allowed the synthesis of cost estimates that included different combinations of cost categories. Under this approach, a regression equation with a separate cost intercept was specified for each of four cost categories: *Labor* (${\widehat{c}}_{i}^{l}$), *Supply chain* (${\widehat{c}}_{i}^{sc}$), *Service delivery* (${\widehat{c}}_{i}^{sd}$), and *Capital* (${\widehat{c}}_{i}^{c}$). Each regression equation estimated the cost per dose for a given cost category as a function of the covariates in Table 1:

**Table 1:** Model covariates

Covariate name	Description
Labor indicator (${I}_{l}$)	Labor cost category included in cost per dose = 1; otherwise = 0
Supply chain indicator (${I}_{sc}$)	Supply chain cost category included in cost per dose = 1; otherwise = 0
Service delivery indicator (${I}_{sd}$)	Service delivery cost category included in cost per dose = 1; otherwise = 0
Capital indicator (${I}_{c}$)	Capital cost category included in cost per dose = 1; otherwise = 0
Year	Study year
Econ	Cost type: financial = 0; economic = 1; undefined = 2
Single	Antigens included: full vaccine program = 0; single antigen = 1
log(Doses)	Number of doses in the routine vaccination schedule in study year, logged
log(GDP)	GDP per capita in study year, logged
DTP3	DTP3 coverage in study year
log(Pop)	Country population size in study year, logged

*DTP3* diphtheria-tetanus-pertussis third dose coverage, *GDP* gross domestic product, *Pop* population

1 $${\widehat{c}}_{i}^{l}=\mathrm{exp}\left({\beta }_{{0}_{l}}+{\beta }_{1}*{Year}_{i}+{\beta }_{2}*{Econ}_{i}+{\beta }_{3}*{Single}_{i}+{\beta }_{4}*{\mathit{log}\left(Doses\right)}_{i}+{\beta }_{5}*{DTP3}_{i}+{\beta }_{6}*{\mathit{log}\left(GDP\right)}_{i}+{\beta }_{7}*{\mathit{log}\left(Pop\right)}_{i}\right)$$

2 $${\widehat{c}}_{i}^{sc}=\mathrm{exp}\left({\beta }_{{0}_{sc}}+{\beta }_{1}*{Year}_{i}+{\beta }_{2}*{Econ}_{i}+{\beta }_{3}*{Single}_{i}+{\beta }_{4}*{\mathit{log}\left(Doses\right)}_{i}+{\beta }_{5}*{DTP3}_{i}+{\beta }_{6}*{\mathit{log}\left(GDP\right)}_{i}+{\beta }_{7}*{\mathit{log}\left(Pop\right)}_{i}\right)$$

3 $${\widehat{c}}_{i}^{sd}=\mathrm{exp}\left({\beta }_{{0}_{sd}}+{\beta }_{1}*{Year}_{i}+{\beta }_{2}*{Econ}_{i}+{\beta }_{3}*{Single}_{i}+{\beta }_{4}*{\mathit{log}\left(Doses\right)}_{i}+{\beta }_{5}*{DTP3}_{i}+{\beta }_{6}*{\mathit{log}\left(GDP\right)}_{i}+{\beta }_{7}*{\mathit{log}\left(Pop\right)}_{i}\right)$$

4 $${\widehat{c}}_{i}^{c}=\mathrm{exp}\left({\beta }_{{0}_{c}}+{\beta }_{1}*{Year}_{i}+{\beta }_{2}*{Econ}_{i}+{\beta }_{3}*{Single}_{i}+{\beta }_{4}*{\mathit{log}\left(Doses\right)}_{i}+{\beta }_{5}*{DTP3}_{i}+{\beta }_{6}*{\mathit{log}\left(GDP\right)}_{i}+{\beta }_{7}*{\mathit{log}\left(Pop\right)}_{i}\right)$$

An additional equation related these cost category unit costs to the combination of cost categories included in each empirical study estimate, with ${tc}_{i}$ representing the mean estimate of the delivery cost per dose for a given study:

5 $${tc}_{i} ={\widehat{c}}_{i}^{l}*{I}_{l}+{\widehat{c}}_{i}^{sc}*{I}_{sc}+{\widehat{c}}_{i}^{sd}*{I}_{sd}+{\widehat{c}}_{i}^{c}*{I}_{c}$$

These ${tc}_{i}$ values were used to parameterize a Gamma likelihood function for the observed data (${y}_{i}$), where the shape parameter $\alpha$ described the residual variance:

6 $${y}_{i} \sim Gamma\left(\alpha ,\frac{\alpha }{{tc}_{i}}\right)$$

This specification assumed variance proportional to ${tc}_{i}$. We assumed informative prior distributions for all regression coefficients, which were assumed to follow a normal distribution centered at zero with a standard deviation of 1 [15]. The shape parameter $\alpha$ was assumed to follow a half-Cauchy distribution centered at zero with a standard deviation of 5 [16]. The prediction model was estimated in R software [17] using an adaptive Hamiltonian Monte Carlo algorithm using the Stan software package, version 2.20.0, with four chains of 5000 iterations. The first 2500 iterations were discarded (burn-in period), yielding 10,000 posterior draws for analysis [18, 19]. Stan model diagnostics were utilized to determine any problems encountered by the sampler, and the potential scale reduction factor (i.e., Rhat) for all parameters was evaluated to determine that the model had successfully converged.

The fitted prediction model was used to generate both economic and financial programmatic delivery cost per dose estimates (i.e., as opposed to generating single antigen cost per dose estimates) for each LMIC for 2009–2018. To generate these estimates, we predicted values from the fitted model, with covariates values specific to each country and year. Estimates included all cost categories and are presented in 2018 US dollars, inflated to 2018 values in local currency using the country consumer price index, and converted to 2018 US dollars using market exchange rates [12]. Global and regional cost per dose estimates were calculated as population-weighted averages of individual country estimates. We tested predictive performance by comparing model predictions to the observed cost per dose matched to country and year.

### Sensitivity Analysis: Alternative Regression Specifications

We estimated several alternative regression specifications as robustness checks. First, we re-estimated the model having excluded two outlier observations with cost per dose estimates less than $0.01 as a robustness check. Second, we adopted weakly informative prior distributions, i.e., all predictors were assumed to follow a Normal distribution centered at zero with a standard deviation of 10 instead of 1. Finally, we adopted non-informative prior distributions for parameters.

## Results

### Data

A total of 52 routine delivery cost per dose estimates (i.e., excluding vaccine costs) from 29 studies covering 24 countries were included in the analysis [20–48]. For example, a study may have included both economic and financial cost per dose estimates, which were both included in this analysis. Of the 29 studies, 13 were undertaken in low-income country settings and 16 in middle-income country settings, as classified by 2019 World Bank income level [10]. The observed cost per dose ranged from $0.66 to $9.45 (focusing on studies that included all cost categories). Thirty-four unit cost estimates (65%) could not be disaggregated into separate cost categories; the remaining observations could be disaggregated into unique categories, resulting in a total of 119 unique observations included in the analysis. When examining the total cost per dose by cost categories included, 92% of observations included labor, 98% supply chain, 63% service delivery, and 63% capital. Table 2 provides summary information on the empirical studies used in the analysis. For the observed dataset, the mean and standard deviation (in parentheses) of the continuous explanatory variables were 12 (3) for *Doses*, $1550 ($1300) for *GDP*, 0.88 (0.09) for *DTP3*, and 57,100,000 (207,000,000) for *Pop*.

**Table 2:** Summary characteristics for immunization delivery unit cost per dose data

	Estimates (*n*)
Reported cost per dose	
Total cost per dose only	34
Total + cost categories	18^a^
Income^b^	
Low income	32
Lower middle income	16
Upper middle income	4
Antigens costed	
Single antigen	28
Full vaccination program	24
Cost type	
Economic	27
Financial	11
Undefined	14

^a^There were 52 total cost per dose observations with reported base years between 2001 and 2017, but the 18 cost per dose estimates that could be disaggregated into cost categories brought the total analyzed observations from 52 to 119

^b^Low income: gross national income (GNI) per capita of $1025 or less; lower middle income: GNI per capita of $1026–$3995; upper middle income: GNI per capita of $3996–$12,375 [12]. Costs in US dollars

### Regression Model

Table 3 reports point estimates and standard errors for regression coefficients and other model parameters. While certain cost category intercepts, the single antigen indicator, and the coefficients on log(*Pop*) and DTP3 were statistically significantly different from zero, the coefficients for other non-intercept predictor variables were not significant.

**Table 3:** Results for regressions of routine childhood delivery unit cost per dose on predictors

Variable	Mean coefficient
Cost category intercepts	
Labor	0.08 (0.23)
Supply chain	− 0.97 (0.26)
Service delivery	− 0.07 (0.28)
Capital	− 1.16 (0.31)
Predictors	
Year	− 0.14 (0.13)
Economic cost indicator	− 0.06 (0.14)
Single antigen indicator	− 0.49 (0.22)*
log(Doses)	0.01 (0.13)
log(GDP per capita)	0.19 (0.13)
log(Pop)	− 0.30 (0.12)*
DTP3 coverage	0.29 (0.12)*
Gamma dispersion parameter	
Alpha	1.08 (0.07)

Continuous predictors were standardized to mean zero and unit standard deviation; thus, fitted coefficients for continuous variables [e.g., log(Doses)] represent the increase in log cost per dose observed for a 1.0 standard deviation increase in the variable. Values in parentheses represent standard errors

*DTP3* diphtheria-tetanus-pertussis third dose coverage, *GDP* gross domestic product, *Pop* population

^*^Significant at 5% level

**Fig. 1:**
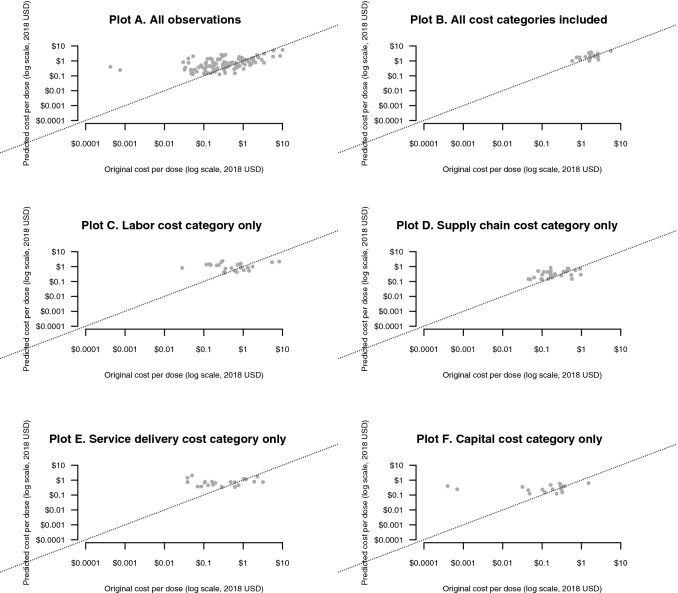
Comparison of predicted cost per dose and published literature cost per dose for routine childhood vaccine delivery. The original costs per dose represent 119 observations across 24 countries for reported base years between 2001 and 2017. The predicted costs per dose are matched to the country and year of each observation

We assessed in-sample fit by comparing observed versus predicted values for the study sample (Fig. 1). Figure 1 includes six subplots: (A) includes all observations; (B) includes observations in which all four cost categories were included in the total cost per dose; (C) includes observations that contained the labor cost category only; (D) includes observations that contained the supply chain category only; (E) includes observations that contained the service delivery cost category only; and (F) includes observations that contained the capital cost category only.

We calculated first differences to describe the percentage difference in the cost per dose associated with specified changes in individual predictors, holding others fixed. These values represent posterior means, and values in parentheses represent equal-tailed 95% credible intervals. More recent studies reported lower cost per dose estimates, controlling for other covariates. The routine cost per dose was estimated to decline by 4.6% (− 4.2%, 13.0%) for each additional calendar year, increase by 0.2% (− 3.7%, 3.9%) for each additional dose added to the routine vaccination schedule, increase by 21.1% (− 5.3%, 52.5%) when GDP per capita was two times greater, decline by 14.1% (3.0%, 24.0%) when population size was two times greater, and increase by 3.4% (0.7%, 6.0%) for each percentage point increase in DTP3.

### Estimated Costs Per Dose for All LMICs

**Fig. 2:**
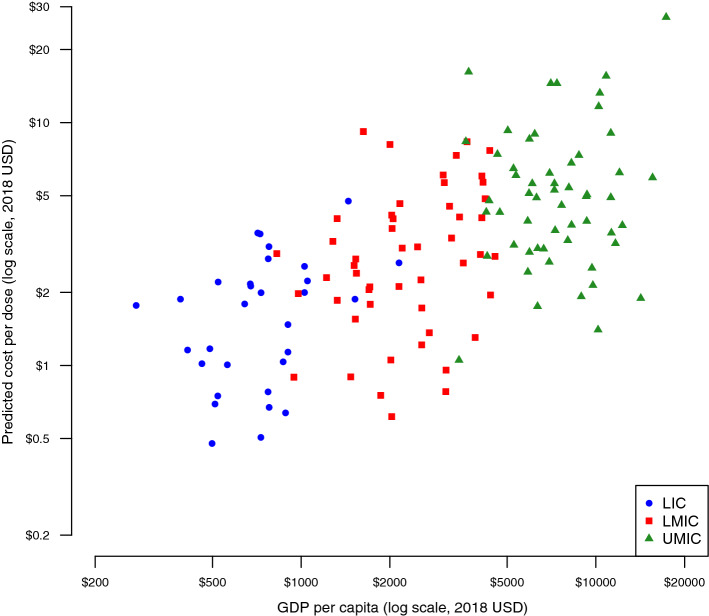
Predicted economic cost per dose in 2018 for routine childhood vaccine delivery by GDP per capita and World Bank income level for 136 LIC and middle-income countries. *GDP* gross domestic product, *LIC* low-income countries, *LMIC* lower middle-income countries, *UMIC* upper middle-income countries

For the year 2018, the population-weighted average economic cost per dose for routine delivery of childhood vaccines was estimated to be $1.87 (95% uncertainty interval $0.64–4.38) across all LMICs. By individual cost category, the programmatic economic cost per dose for routine delivery of childhood vaccines was $0.74 ($0.26–1.70) for labor, $0.26 ($0.08–0.67) for supply chain, $0.22 ($0.06–0.57) for capital, and $0.65 ($0.20–1.66) for other service delivery costs. By income level, the average predicted programmatic, economic cost per dose was $1.41 ($0.52–3.16) for low-income countries, $1.36 ($0.44–3.32) for lower middle-income countries, and $2.59 ($0.82–6.38) for upper middle-income countries. Figure 2 presents the country-level cost per dose estimates by GDP per capita and World Bank income level for 136 LMICs. Table 4 presents the programmatic, economic cost per dose by each stratification by world region and income level.

**Table 4:** Predicted economic cost per dose (US$) in 2018 for routine childhood vaccine delivery by cost category and world region/income level

	Total cost per dose	Labor cost per dose	Supply chain cost per dose	Service delivery cost per dose	Capital cost per dose
Region					
Africa	$1.49 ($0.57–3.31)	$0.59 ($0.23–1.28)	$0.21 ($0.07–0.49)	$0.52 ($0.17–1.23)	$0.17 ($0.06–0.43)
Americas	$2.61 ($0.87–6.33)	$1.02 ($0.35–2.42)	$0.37 ($0.11–0.94)	$0.91 ($0.27–2.44)	$0.31 ($0.09–0.81)
Eastern Mediterranean	$1.86 ($0.71–4.05)	$0.73 ($0.29–1.56)	$0.26 ($0.09–0.62)	$0.65 ($0.22–1.52)	$0.22 ($0.07–0.52)
Europe	$3.51 ($1.21–8.18)	$1.38 ($0.50–3.19)	$0.50 ($0.15–1.25)	$1.22 ($0.38–3.06)	$0.41 ($0.12–1.07)
Southeast Asia	$1.35 ($0.40–3.48)	$0.53 ($0.16–1.34)	$0.19 ($0.05–0.52)	$0.47 ($0.13–1.26)	$0.16 ($0.04–0.45)
Western Pacific	$2.07 ($0.59–5.47)	$0.81 ($0.24–2.10)	$0.29 ($0.07–0.81)	$0.73 ($0.18–2.09)	$0.24 ($0.06–0.69)
Income level					
Low income	$1.41 ($0.52–3.16)	$0.56 ($0.20–1.24)	$0.20 ($0.07–0.47)	$0.49 ($0.16–1.17)	$0.16 ($0.05–0.41)
Lower middle income	$1.36 ($0.44–3.32)	$0.54 ($0.18–1.28)	$0.19 ($0.06–0.50)	$0.47 ($0.14–1.21)	$0.16 ($0.04–0.43)
Upper middle income	$2.59 ($0.82–6.38)	$1.01 ($0.34–2.44)	$0.37 ($0.10–0.95)	$0.91 ($0.25–2.39)	$0.30 ($0.08–0.81)

Countries included in each World Health Organization (WHO) region are low- and middle-income countries according to World Bank income level in 2019 [12]

**Fig. 3:**
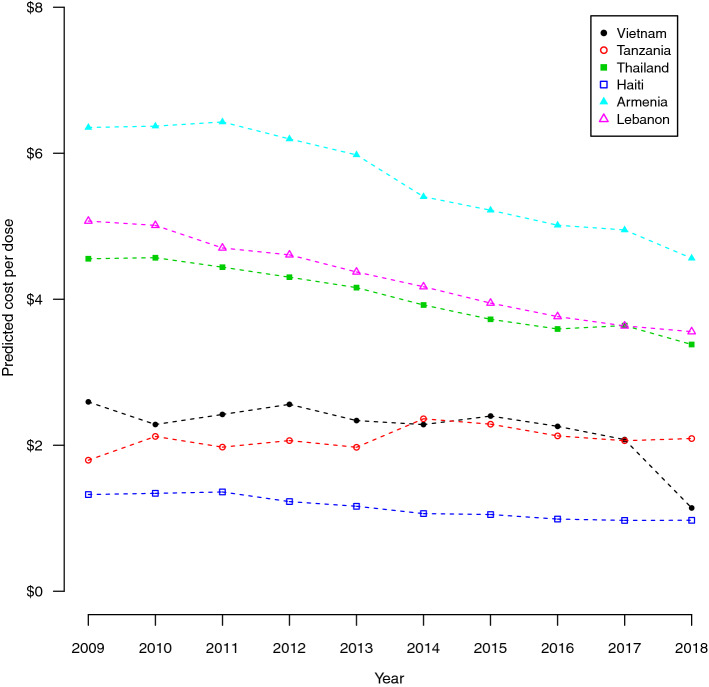
Predicted programmatic, economic cost per dose for routine childhood vaccine delivery by year. Armenia = European region, lower middle income; Haiti = region of the Americas, low income; Lebanon = Eastern Mediterranean region, upper middle income; Tanzania = African region, low income; Thailand = Southeast Asian region, upper middle income; Vietnam = Western Pacific region, lower middle income

The set of country-specific economic cost estimates for delivery cost per dose can be found in Appendix Table A (see the “Supplementary Appendix” in the electronic supplementary material). Figure 3 presents the predicted programmatic, economic cost per dose for childhood vaccine delivery by year for six example countries selected for differences in region and income level. The predicted cost per dose shows a decreasing trend on average over time. Within individual cost categories, financial cost per dose estimates did not differ substantially from the economic cost per dose predictions; however, financial cost observations generally reported fewer cost categories, notably capital. The set of country-specific financial cost estimates for delivery cost per dose can be found in Appendix Table B. Appendix Table C further provides the median and interquartile range globally, and by region and income level.

### Sensitivity Analysis: Alternative Regression Specifications

With the first alternative regression specification, excluding two outliers produced small changes in most coefficients and large changes in a few (Appendix Table D) and resulted in cost per dose estimates that were 16% higher on average (Appendix Table E). Adopting weakly informative (Appendix Table F) or non-informative (Appendix Table G) priors did not substantially change regression results (i.e., less than 10% average change in cost per dose estimates).

## Discussion

For the year 2018, the average economic cost per dose across all LMICs for routine delivery of childhood vaccines was estimated to be $1.87 (95% uncertainty interval $0.64–4.38), excluding vaccine costs. By country income classification, the average cost per dose was $1.41 ($0.52–3.16) for low-income countries, $1.36 ($0.44–3.32) for lower middle-income countries, and $2.59 ($0.82–6.38) for upper middle-income countries. These estimates are consistent with the empirical estimates reported in the ICAN IDCC [11], where studies including all cost categories averaged $1.41 for low-income countries and $4.02 for lower middle-income countries (there was only one empirical estimate, $1.87, for upper middle-income countries). While the population-weighted average predicted cost per dose estimates were similar for low- and lower middle-income countries, Fig. 2 indicates that costs were predicted to be higher for richer countries overall. Costs were also predicted to be lower for more populous countries (e.g., Ethiopia and Nigeria), compared to similar countries with smaller populations.

These predicted cost per dose estimates can be useful for cost-effectiveness analyses when country-level costs are unavailable, highly uncertain, or old. For example, instead of using neighboring country data or regional data when primary cost data are unavailable—or creating estimates based on expert opinion alone—these modeled costs provide another alternative to use, based on a broader set of data. While recent costing reference cases provide concrete guidance for implementing and reporting costing studies [49, 50], we are unlikely to have the resources to conduct empirical costing and/or cost-effectiveness analysis for all questions and settings of interest. Therefore, a key strategy for improving the availability of costing data is to determine how and where we can leverage insights from specific studies to understand a general theme. Country-specific costs modeled within a Bayesian meta-regression framework provide a broad indication of immunization delivery costs that may be useful when accurate local data are unavailable.

The regression results showed several relationships that might be expected between predictors and immunization delivery costs. The statistically significant relationships were population size and DTP3; higher population size (a proxy for higher service volume at the site level) was associated with lower cost per dose, while DTP3 (a proxy for overall coverage of the immunization program) was associated with higher cost per dose. While the reason for the significant relationship with population size is unclear, it could be related to economics of scale, i.e., the increasing scale of an immunization program results in cost savings through both efficiency gains and spreading fixed costs over a larger population. Additionally, we did not investigate alternative measures of service volume, such as under-five population, which may impact the results. However, we expect that the direction of this relationship is likely to remain the same, as the higher service volume to lower delivery costs relationship has also been found in previous studies [8]. Higher DTP3 being associated with higher cost per dose may be due to increasing marginal costs with higher vaccine coverage levels, or some other feature of higher-coverage programs that leads to higher costs. Greater GDP per capita (a proxy for country price levels) was also associated with higher cost per dose. Additionally, we found no relationship between number of doses in the routine immunization schedule and unit cost, although we might expect to observe a lower cost per dose due to economies of scale. We also found the sign estimated for some coefficients was different to what might have been expected (e.g., the negative signs on *Year* and the economic cost indicator, *Econ*). If interpreted directly, these findings would suggest lower costs (in real terms) in more recent years, holding other covariates constant, and economic costs which are lower than financial cost estimates. However, the statistical precision of these two findings (as expressed by the *p* values) is low, and consequently little weight should be placed on the point estimates. In addition, the direction and magnitude of the coefficients in the model could be driven by unobserved characteristics of the included studies (e.g., the costs of inputs or the data collection approach) that are correlated with both the predictor variables and the unit cost. For this reason, all of these relationships should be viewed as correlations with the predicted cost per dose and should not be interpreted causally.

There are several limitations to this analysis. First, the studies we included in the analysis were heterogeneous in terms of the scope of costing, site selection, data collection methods, and the level of detail with which results were reported. This heterogeneity in costing study methods and reporting has been observed by prior reviews [51–55]. We attempted to deal with this by adopting an analytic strategy that allowed for differences in the cost categories and types of costs (i.e., financial vs. economic) reported. However, it is unlikely our approach fully reconciled all methodological differences between studies. For this reason, the residual variance of the meta-regression model—which was substantial—will reflect not only sampling uncertainty but also non-sampling error due to methodological heterogeneity, such as inconsistent definitions of cost categories and cost types. This could induce omitted-variable bias, if methodological differences were correlated with regression variables, or if cost estimates were biased systematically. For example, it has been observed that costing studies may over- or underestimate costs due to the costing approach used (i.e., gross costing vs. micro-costing [56]) or may underestimate costs due to the exclusion of relevant intervention cost categories [57]. In our analysis, we saw that excluding outliers changed the magnitude, although not the direction, of several regression coefficients, as shown in the results of Appendix Table C. All included studies were reported from the immunization provider perspective, and excluded caregiver/beneficiary time and transportation costs to receive vaccination. As the definition of this provider perspective may have varied across studies, we defined cost categories to include the same elements in order to improve comparability. However, for categories that included both recurrent and investment costs, the treatment of investment costs (e.g., useful life estimates, annuitization) may differ between individual costing studies. A second limitation stems from the assumption that routine childhood vaccine delivery costs are similar regardless of individual vaccine product. This assumption was necessitated by the format in which data were reported, yet in reality there may be differences in delivery costs, particularly in the case of injection versus oral vaccines that involve differences in training and delivery, or where multiple doses are delivered in a single immunization visit. Furthermore, as routine immunization schedules were not available for all historical years, country-specific time trends in the number of doses in the immunization schedule (represented by the *Doses* variable) may be less accurate for earlier years. A third limitation relates to sample size. While we were able to include a large number of studies in this meta-regression analysis, the average number of observations within each of these studies (i.e., number of sites) was small. A costing study that relies on a small number of sampled sites may produce results that are not representative, if there is likely to be large variation across sites [58]. In addition, the 24 countries included in the dataset were not a representative sample of all LMICs. The countries represented by the sampled studies were generally of lower GDP per capita (average $1600 vs. $4000) and higher DTP3 (average coverage 88% vs. 85%) compared to the 136 LMICs for which we provide modeled estimates. While we adjusted for these factors in the meta-regression model, the imperfect overlap of sample and population adds to the uncertainty in country-level unit costs estimated for countries with higher GDP per capita, lower population size, and lower DTP3. Therefore, estimates will be less reliable for countries with combinations of covariates not included in the analyzed sample, as shown by the estimates for countries such as Tuvalu and Palau (Appendix Table A).

In light of the issues described above, this analysis does not fully resolve the evidence gap created by the limited number of immunization costing studies that are published (limited relative to the number of settings and policy questions where they would be useful), and the estimates we report inherit many of the limitations of the empirical studies they are based on. Additional primary data collection on delivery costs of programs is necessary. However, the need to make policy choices based on imperfect information is unavoidable, and the estimates we report provide an additional evidence source for analysts missing this important input to their analysis.

Immunization delivery costs are a necessary category of high-quality cost-effectiveness models, and are also used to inform resource mobilization for immunization programs. Using observed costs per dose from 29 studies in 24 countries, our study provides estimates for 136 LMICs for the years 2009–2018 produced via meta-regression analyses that can help improve resource mobilization and planning in situations where empirical cost data are unavailable or of low quality. This methodology has the potential to be applied to many other areas of health care for LMICs in which it is unlikely that primary data cost studies would be conducted for all settings/services of interest.

## Electronic supplementary material

Below is the link to the electronic supplementary material.

Supplementary file1 (DOCX 44 kb)

## Data Availability

The data that support the findings of this study are openly available in the Immunization Delivery Cost Catalogue (IDCC) at https://immunizationeconomics.org/ican-idcc/, reference number [11].
