# Supplementary material for: Producing Standardized Country-Level Immunization Delivery Unit Cost Estimates
Source: Pharmacoeconomics. 2020 Jun 28;38(9):995–1005. doi: 10.1007/s40273-020-00930-6 (PMC7437655; doi:10.1007/s40273-020-00930-6)
Supplement: Supplementary file 1 — Supplementary file1 (DOCX 44 kb) [file 40273_2020_930_MOESM1_ESM.docx]

**Supplementary Appendix**

**Appendix Table A. Low- and middle-income country parameters with predicted programmatic, economic immunization delivery cost per dose in 2018.**

| **Country** | **World Bank Income Level^a^ [10]** | **WHO Region** | **GDP per capita**  **(2018 US$) [11]** | **Population [11]** | **DTP3 Coverage [12]** | **Number of Doses in Schedule^b^** | **Predicted Economic Delivery Cost per Dose for Childhood Vaccines** |
| --- | --- | --- | --- | --- | --- | --- | --- |
| Afghanistan | LIC | EMR | $520 | 37,200,000 | 0.66 | 18 | $0.75 ($0.20–2.02) |
| Albania | UMIC | EUR | $5,250 | 2,870,000 | 0.99 | 11 | $6.47 ($2.90–12.78) |
| Algeria | UMIC | AFR | $4,280 | 42,200,000 | 0.91 | 22 | $2.82 ($0.89–6.82) |
| Angola | UMIC | AFR | $3,430 | 30,800,000 | 0.59 | 11 | $1.05 ($0.24–3.13) |
| Argentina | UMIC | AMR | $11,650 | 44,500,000 | 0.86 | 18 | $3.18 ($0.85–8.55) |
| Armenia | LMIC | EUR | $4,210 | 2,950,000 | 0.92 | 16 | $4.86 ($2.20–9.59) |
| Azerbaijan | UMIC | EUR | $4,720 | 9,940,000 | 0.95 | 17 | $4.27 ($1.81–8.65) |
| Bangladesh | LMIC | SEAR | $1,700 | 161,000,000 | 0.98 | 18 | $2.05 ($0.65–5.04) |
| Belarus | UMIC | EUR | $6,290 | 9,490,000 | 0.97 | 13 | $4.91 ($2.18–9.72) |
| Belize | UMIC | AMR | $5,030 | 383,000 | 0.96 | 12 | $9.28 ($3.71–20.10) |
| Benin | LIC | AFR | $900 | 11,500,000 | 0.76 | 18 | $1.47 ($0.52–3.38) |
| Bhutan | LMIC | SEAR | $3,360 | 754,000 | 0.97 | 13 | $7.33 ($3.15–14.97) |
| Bolivia | LMIC | AMR | $3,550 | 11,400,000 | 0.83 | 18 | $2.64 ($1.00–5.81) |
| Bosnia and Herzegovina | UMIC | EUR | $5,950 | 3,320,000 | 0.73 | 12 | $2.93 ($0.95–7.21) |
| Botswana | UMIC | AFR | $8,260 | 2,250,000 | 0.95 | 13 | $6.82 ($2.98–13.85) |
| Brazil | UMIC | AMR | $8,920 | 209,000,000 | 0.83 | 14 | $1.92 ($0.48–5.38) |
| Bulgaria | UMIC | EUR | $9,270 | 7,020,000 | 0.92 | 11 | $4.97 ($2.07–10.56) |
| Burkina Faso | LIC | AFR | $730 | 19,800,000 | 0.91 | 12 | $1.99 ($0.74–4.44) |
| Burundi | LIC | AFR | $280 | 11,200,000 | 0.90 | 16 | $1.77 ($0.55–4.37) |
| Cabo Verde | LMIC | AFR | $3,650 | 544,000 | 0.98 | 15 | $8.35 ($3.61–16.83) |
| Cambodia | LMIC | WPR | $1,510 | 16,200,000 | 0.92 | 18 | $2.58 ($1.06–5.36) |
| Cameroon | LMIC | AFR | $1,530 | 25,200,000 | 0.79 | 9 | $1.55 ($0.53–3.61) |
| Central African Republic | LIC | AFR | $510 | 4,670,000 | 0.47 | 14 | $0.69 ($0.12–2.29) |
| Chad | LIC | AFR | $730 | 15,500,000 | 0.41 | 17 | $0.51 ($0.08–1.80) |
| China | UMIC | WPR | $9,770 | 1,393,000,000 | 0.99 | 7 | $2.14 ($0.55–5.85) |
| Colombia | UMIC | AMR | $6,650 | 49,600,000 | 0.92 | 13 | $3.02 ($1.11–6.66) |
| Comoros | LIC | AFR | $1,450 | 832,000 | 0.91 | 17 | $4.75 ($1.99–9.77) |
| Congo | LMIC | AFR | $2,150 | 5,200,000 | 0.75 | 16 | $2.11 ($0.77–4.73) |
| Congo, Democratic Republic of the | LIC | AFR | $560 | 84,100,000 | 0.81 | 16 | $1.01 ($0.32–2.47) |
| Costa Rica | UMIC | AMR | $12,030 | 5,000,000 | 0.94 | 14 | $6.23 ($2.48–13.57) |
| Côte d'Ivoire | LMIC | AFR | $1,720 | 25,100,000 | 0.82 | 19 | $1.79 ($0.65–4.07) |
| Cuba | UMIC | AMR | $8,100 | 11,300,000 | 0.99 | 12 | $5.40 ($2.34–10.84) |
| Djibouti | LMIC | EMR | $2,050 | 959,000 | 0.84 | 16 | $4.02 ($1.66–8.38) |
| Dominica | UMIC | AMR | $7,030 | 71,600 | 0.94 | 11 | $14.52 ($4.42–36.62) |
| Dominican Republic | UMIC | AMR | $7,650 | 10,600,000 | 0.94 | 9 | $4.57 ($1.91–9.52) |
| Ecuador | UMIC | AMR | $6,340 | 17,100,000 | 0.85 | 16 | $3.03 ($1.09–6.91) |
| Egypt | LMIC | EMR | $2,550 | 98,400,000 | 0.95 | 16 | $2.25 ($0.79–5.09) |
| El Salvador | LMIC | AMR | $4,060 | 6,420,000 | 0.81 | 10 | $2.86 ($1.08–6.37) |
| Equatorial Guinea | UMIC | AFR | $10,170 | 1,310,000 | 0.25 | 16 | $1.40 ($0.09–6.76) |
| Eritrea | LIC | AFR | $730 | 3,180,000 | 0.95 | 10 | $3.48 ($1.14–8.52) |
| Ethiopia | LIC | AFR | $770 | 109,000,000 | 0.72 | 13 | $0.78 ($0.22–2.03) |
| Fiji | UMIC | WPR | $6,200 | 883,000 | 0.99 | 19 | $8.98 ($3.90–18.34) |
| Gabon | UMIC | AFR | $8,030 | 2,120,000 | 0.70 | 9 | $3.28 ($0.89–8.85) |
| Gambia | LIC | AFR | $710 | 2,280,000 | 0.93 | 10 | $3.51 ($1.12–8.68) |
| Georgia | UMIC | EUR | $4,340 | 3,730,000 | 0.93 | 11 | $4.77 ($2.12–9.54) |
| Ghana | LMIC | AFR | $2,200 | 29,800,000 | 0.97 | 5 | $3.04 ($0.90–7.94) |
| Grenada | UMIC | AMR | $10,830 | 111,000 | 0.96 | 11 | $15.56 ($5.08–38.42) |
| Guatemala | LMIC | AMR | $4,550 | 17,200,000 | 0.86 | 15 | $2.81 ($1.09–6.08) |
| Guinea | LIC | AFR | $890 | 12,400,000 | 0.45 | 7 | $0.64 ($0.09–2.37) |
| Guinea-Bissau | LIC | AFR | $780 | 1,870,000 | 0.88 | 17 | $3.09 ($1.21–6.56) |
| Guyana | UMIC | AMR | $4,630 | 779,000 | 0.95 | 14 | $7.41 ($3.26–14.9) |
| Haiti | LIC | AMR | $870 | 11,100,000 | 0.64 | 18 | $1.04 ($0.29–2.74) |
| Honduras | LMIC | AMR | $2,480 | 9,590,000 | 0.90 | 18 | $3.08 ($1.31–6.29) |
| India | LMIC | SEAR | $2,020 | 1,353,000,000 | 0.89 | 14 | $1.05 ($0.25–2.96) |
| Indonesia | LMIC | SEAR | $3,890 | 267,700,000 | 0.79 | 18 | $1.30 ($0.31–3.71) |
| Iran | UMIC | EMR | $5,270 | 81,800,000 | 0.99 | 8 | $3.13 ($1.21–6.68) |
| Iraq | UMIC | EMR | $5,880 | 38,400,000 | 0.84 | 15 | $2.43 ($0.82–5.66) |
| Jamaica | UMIC | AMR | $5,360 | 2,930,000 | 0.97 | 13 | $6.07 ($2.8–11.72) |
| Jordan | UMIC | EMR | $4,250 | 9,960,000 | 0.96 | 17 | $4.28 ($1.84–8.6) |
| Kazakhstan | UMIC | EUR | $9,330 | 18,300,000 | 0.98 | 16 | $5.05 ($1.87–11.09) |
| Kenya | LMIC | AFR | $1,710 | 51,400,000 | 0.92 | 18 | $2.11 ($0.78–4.69) |
| Kiribati | LMIC | WPR | $1,630 | 116,000 | 0.95 | 12 | $9.19 ($2.70–23.06) |
| Korea, Democratic People’s Republic of | LIC | SEAR | $1,030 | 25,500,000 | 0.97 | 7 | $2.56 ($0.80–6.39) |
| Kyrgyz Republic | LMIC | EUR | $1,280 | 6,320,000 | 0.94 | 10 | $3.24 ($1.26–7.08) |
| Lao People’s Democratic Republic | LMIC | WPR | $2,570 | 7,060,000 | 0.68 | 19 | $1.72 ($0.52–4.41) |
| Lebanon | UMIC | EMR | $8,270 | 6,850,000 | 0.83 | 18 | $3.79 ($1.29–9.00) |
| Lesotho | LMIC | AFR | $1,320 | 2,110,000 | 0.93 | 13 | $4.02 ($1.66–8.45) |
| Liberia | LIC | AFR | $670 | 4,820,000 | 0.84 | 16 | $2.12 ($0.80–4.62) |
| Libya | UMIC | EMR | $7,240 | 6,680,000 | 0.97 | 17 | $5.61 ($2.33–11.51) |
| Macedonia, North | UMIC | EUR | $6,080 | 2,080,000 | 0.91 | 14 | $5.61 ($2.45–11.35) |
| Madagascar | LIC | AFR | $460 | 26,300,000 | 0.75 | 17 | $1.02 ($0.32–2.49) |
| Malawi | LIC | AFR | $390 | 18,100,000 | 0.92 | 9 | $1.87 ($0.52–4.98) |
| Malaysia | UMIC | WPR | $11,240 | 31,500,000 | 0.99 | 15 | $4.92 ($1.70–11.24) |
| Maldives | UMIC | SEAR | $10,220 | 516,000 | 0.99 | 17 | $11.66 ($4.73–25.04) |
| Mali | LIC | AFR | $900 | 19,100,000 | 0.71 | 16 | $1.13 ($0.36–2.76) |
| Marshall Islands | UMIC | WPR | $3,620 | 58,400 | 0.81 | 16 | $8.37 ($2.50–21.18) |
| Mauritania | LMIC | AFR | $1,220 | 4,400,000 | 0.81 | 10 | $2.30 ($0.79–5.37) |
| Mauritius | UMIC | AFR | $11,240 | 1,270,000 | 0.97 | 10 | $9.06 ($3.67–19.78) |
| Mexico | UMIC | AMR | $9,700 | 126,000,000 | 0.88 | 14 | $2.52 ($0.72–6.53) |
| Micronesia | LMIC | WPR | $3,060 | 113,000 | 0.75 | 19 | $5.66 ($1.71–14.12) |
| Moldova, Republic of | LMIC | EUR | $3,190 | 3,550,000 | 0.93 | 20 | $4.53 ($1.98–9.01) |
| Mongolia | LMIC | WPR | $4,100 | 3,170,000 | 0.99 | 19 | $6.03 ($2.67–11.87) |
| Montenegro | UMIC | EUR | $8,760 | 622,000 | 0.87 | 9 | $7.34 ($2.51–17.56) |
| Morocco | LMIC | EMR | $3,240 | 36,000,000 | 0.99 | 16 | $3.35 ($1.32–7.03) |
| Mozambique | LIC | AFR | $490 | 29,500,000 | 0.80 | 15 | $1.17 ($0.39–2.78) |
| Myanmar | LMIC | SEAR | $1,330 | 53,700,000 | 0.91 | 9 | $1.85 ($0.68–4.19) |
| Namibia | UMIC | AFR | $5,930 | 2,450,000 | 0.89 | 18 | $5.12 ($2.12–10.76) |
| Nepal | LIC | SEAR | $1,030 | 28,100,000 | 0.91 | 13 | $2.00 ($0.79–4.3) |
| Nicaragua | LMIC | AMR | $2,030 | 6,470,000 | 0.98 | 8 | $4.16 ($1.56–9.38) |
| Niger | LIC | AFR | $410 | 22,400,000 | 0.79 | 16 | $1.16 ($0.37–2.77) |
| Nigeria | LMIC | AFR | $2,030 | 196,000,000 | 0.57 | 18 | $0.62 ($0.11–2.05) |
| Pakistan | LMIC | EMR | $1,470 | 212,000,000 | 0.75 | 16 | $0.90 ($0.24–2.41) |
| Palau | UMIC | WPR | $17,320 | 17,900 | 0.95 | 14 | $27.17 ($7.10–75.69) |
| Panama | UMIC | AMR | $15,580 | 4,180,000 | 0.88 | 17 | $5.94 ($1.96–14.51) |
| Papua New Guinea | LMIC | WPR | $2,720 | 8,610,000 | 0.61 | 13 | $1.36 ($0.35–3.78) |
| Paraguay | UMIC | AMR | $5,870 | 6,960,000 | 0.88 | 17 | $3.94 ($1.56–8.45) |
| Peru | UMIC | AMR | $6,950 | 32,000,000 | 0.84 | 16 | $2.67 ($0.87–6.43) |
| Philippines | LMIC | WPR | $3,100 | 107,000,000 | 0.65 | 16 | $0.96 ($0.22–2.86) |
| Romania | UMIC | EUR | $12,300 | 19,500,000 | 0.86 | 17 | $3.78 ($1.13–9.5) |
| Russian Federation | UMIC | EUR | $11,290 | 144,000,000 | 0.97 | 17 | $3.52 ($0.9–9.34) |
| Rwanda | LIC | AFR | $770 | 12,300,000 | 0.97 | 19 | $2.75 ($1.07–5.94) |
| Samoa | LMIC | WPR | $4,390 | 196,000 | 0.34 | 18 | $1.95 ($0.19–8.07) |
| São Tomé and Principe | LMIC | AFR | $2,000 | 211,000 | 0.95 | 18 | $8.13 ($3.17–17.51) |
| Senegal | LIC | AFR | $1,520 | 15,900,000 | 0.81 | 23 | $1.88 ($0.65–4.36) |
| Serbia | UMIC | EUR | $7,230 | 6,980,000 | 0.96 | 8 | $5.28 ($2.13–11.21) |
| Sierra Leone | LIC | AFR | $520 | 7,650,000 | 0.90 | 20 | $2.21 ($0.81–4.87) |
| Solomon Islands | LMIC | WPR | $2,160 | 653,000 | 0.85 | 12 | $4.64 ($1.71–10.34) |
| Somalia | LIC | EMR | $500 | 15,000,000 | 0.42 | 10 | $0.48 ($0.07–1.72) |
| South Africa | UMIC | AFR | $6,340 | 57,800,000 | 0.74 | 18 | $1.75 ($0.44–4.91) |
| South Sudan | LIC | AFR | $780 | 11,000,000 | 0.49 | 15 | $0.67 ($0.13–2.13) |
| Sri Lanka | LMIC | SEAR | $4,100 | 21,700,000 | 0.99 | 20 | $4.06 ($1.52–8.88) |
| St. Lucia | UMIC | AMR | $10,320 | 182,000 | 0.95 | 21 | $13.25 ($4.85–30.47) |
| St. Vincent and the Grenadines | UMIC | AMR | $7,380 | 110,000 | 0.97 | 24 | $14.52 ($5.36–33.03) |
| Sudan | LMIC | EMR | $980 | 41,800,000 | 0.93 | 19 | $1.98 ($0.72–4.42) |
| Suriname | UMIC | AMR | $5,950 | 576,000 | 0.95 | 18 | $8.56 ($3.65–17.66) |
| Swaziland | LMIC | AFR | $4,140 | 1,140,000 | 0.90 | 20 | $5.68 ($2.44–11.58) |
| Syrian Arab Republic | LMIC | EMR | $1,860 | 16,900,000 | 0.47 | 18 | $0.75 ($0.13–2.53) |
| Tajikistan | LMIC | EUR | $830 | 9,100,000 | 0.96 | 20 | $2.89 ($1.14–6.16) |
| Tanzania, United Republic of | LIC | AFR | $1,050 | 56,300,000 | 0.98 | 18 | $2.23 ($0.8–5.07) |
| Thailand | UMIC | SEAR | $7,270 | 69,400,000 | 0.97 | 20 | $3.60 ($1.03–9.06) |
| Timor-Leste | LMIC | SEAR | $2,040 | 1,270,000 | 0.83 | 20 | $3.67 ($1.51–7.61) |
| Togo | LIC | AFR | $670 | 7,890,000 | 0.88 | 18 | $2.17 ($0.84–4.67) |
| Tonga | LMIC | WPR | $4,360 | 103,000 | 0.81 | 24 | $7.68 ($2.53–18.28) |
| Tunisia | LMIC | EMR | $3,450 | 11,600,000 | 0.97 | 19 | $4.09 ($1.7–8.39) |
| Turkey | UMIC | EUR | $9,310 | 82,300,000 | 0.98 | 21 | $3.93 ($1.01–10.49) |
| Turkmenistan | UMIC | EUR | $6,970 | 5,850,000 | 0.99 | 20 | $6.19 ($2.44–13.10) |
| Tuvalu | UMIC | WPR | $3,700 | 11,500 | 0.89 | 21 | $16.18 ($4.42–43.19) |
| Uganda | LIC | AFR | $640 | 42,700,000 | 0.93 | 21 | $1.79 ($0.60–4.17) |
| Ukraine | LMIC | EUR | $3,100 | 44,600,000 | 0.50 | 18 | $0.78 ($0.13–2.68) |
| Uzbekistan | LMIC | EUR | $1,530 | 33,000,000 | 0.98 | 19 | $2.74 ($1.05–5.94) |
| Vanuatu | LMIC | WPR | $3,030 | 293,000 | 0.85 | 20 | $6.08 ($2.38–13.25) |
| Venezuela | UMIC | AMR | $14,200 | 28,900,000 | 0.60 | 23 | $1.89 ($0.26–6.84) |
| Vietnam | LMIC | WPR | $2,560 | 95,500,000 | 0.75 | 15 | $1.22 ($0.36–3.12) |
| Yemen | LMIC | EMR | $940 | 28,500,000 | 0.65 | 18 | $0.89 ($0.25–2.40) |
| Zambia | LMIC | AFR | $1,540 | 17,400,000 | 0.90 | 18 | $2.40 ($0.98–5.00) |
| Zimbabwe | LIC | AFR | $2,150 | 14,400,000 | 0.89 | 19 | $2.65 ($1.07–5.58) |

^a^ LIC: Gross national income (GNI) per capita of $1,025 or less; LMIC: GNI per capita of $1,026 to $3,995; UMIC: GNI per capita of $3,996 to $12,375 (World Bank 2019).

^b^ Routine childhood immunization schedule includes vaccines for children aged 0-59 months.

Note: AFR = African region; AMR = Region of the Americas; DTP3 = diphtheria-tetanus-pertussis third dose coverage; EMR = Eastern Mediterranean region; EUR = European region; GDP = gross domestic product; LIC = low-income; LMIC = lower middle-income; SEAR = Southeast Asian region; UMIC = upper middle-income; WPR = Western Pacific region.

**Appendix Table B. Low- and middle-income country parameters with predicted programmatic, financial immunization delivery cost per dose in 2018.**

| **Country** | **World Bank Income Level^a^ [10]** | **WHO Region** | **GDP per capita**  **(2018 US$) [11]** | **Population [11]** | **DTP3 Coverage [12]** | **Number of Doses in Schedule^b^** | **Predicted Financial Delivery Cost per Dose for Childhood Vaccines** |
| --- | --- | --- | --- | --- | --- | --- | --- |
| Afghanistan | LIC | EMR | $520 | 37,200,000 | 0.66 | 18 | $0.81 ($0.20–2.25) |
| Albania | UMIC | EUR | $5,250 | 2,870,000 | 0.99 | 11 | $6.89 ($3.08–14.02) |
| Algeria | UMIC | AFR | $4,280 | 42,200,000 | 0.91 | 22 | $3.01 ($0.92–7.48) |
| Angola | UMIC | AFR | $3,430 | 30,800,000 | 0.59 | 11 | $1.15 ($0.24–3.64) |
| Argentina | UMIC | AMR | $11,650 | 44,500,000 | 0.86 | 18 | $3.42 ($0.88–9.65) |
| Armenia | LMIC | EUR | $4,210 | 2,950,000 | 0.92 | 16 | $5.19 ($2.26–10.60) |
| Azerbaijan | UMIC | EUR | $4,720 | 9,940,000 | 0.95 | 17 | $4.56 ($1.87–9.52) |
| Bangladesh | LMIC | SEAR | $1,700 | 161,000,000 | 0.98 | 18 | $2.18 ($0.68–5.31) |
| Belarus | UMIC | EUR | $6,290 | 9,490,000 | 0.97 | 13 | $5.23 ($2.27–10.78) |
| Belize | UMIC | AMR | $5,030 | 383,000 | 0.96 | 12 | $9.90 ($3.86–21.75) |
| Benin | LIC | AFR | $900 | 11,500,000 | 0.76 | 18 | $1.58 ($0.53–3.80) |
| Bhutan | LMIC | SEAR | $3,360 | 754,000 | 0.97 | 13 | $7.80 ($3.33–16.33) |
| Bolivia | LMIC | AMR | $3,550 | 11,400,000 | 0.83 | 18 | $2.84 ($1.02–6.53) |
| Bosnia and Herzegovina | UMIC | EUR | $5,950 | 3,320,000 | 0.73 | 12 | $3.17 ($0.94–8.31) |
| Botswana | UMIC | AFR | $8,260 | 2,250,000 | 0.95 | 13 | $7.28 ($3.11–15.46) |
| Brazil | UMIC | AMR | $8,920 | 209,000,000 | 0.83 | 14 | $2.07 ($0.49–5.99) |
| Bulgaria | UMIC | EUR | $9,270 | 7,020,000 | 0.92 | 11 | $5.32 ($2.12–11.79) |
| Burkina Faso | LIC | AFR | $730 | 19,800,000 | 0.91 | 12 | $2.12 ($0.76–4.80) |
| Burundi | LIC | AFR | $280 | 11,200,000 | 0.90 | 16 | $1.87 ($0.57–4.68) |
| Cabo Verde | LMIC | AFR | $3,650 | 544,000 | 0.98 | 15 | $8.87 ($3.84–18.24) |
| Cambodia | LMIC | WPR | $1,510 | 16,200,000 | 0.92 | 18 | $2.75 ($1.10–5.84) |
| Cameroon | LMIC | AFR | $1,530 | 25,200,000 | 0.79 | 9 | $1.66 ($0.53–4.01) |
| Central African Republic | LIC | AFR | $510 | 4,670,000 | 0.47 | 14 | $0.76 ($0.12–2.59) |
| Chad | LIC | AFR | $730 | 15,500,000 | 0.41 | 17 | $0.55 ($0.08–2.06) |
| China | UMIC | WPR | $9,770 | 1,393,000,000 | 0.99 | 7 | $2.28 ($0.58–6.36) |
| Colombia | UMIC | AMR | $6,650 | 49,600,000 | 0.92 | 13 | $3.23 ($1.14–7.44) |
| Comoros | LIC | AFR | $1,450 | 832,000 | 0.91 | 17 | $5.06 ($2.07–10.59) |
| Congo | LMIC | AFR | $2,150 | 5,200,000 | 0.75 | 16 | $2.28 ($0.78–5.40) |
| Congo, Democratic Republic of the | LIC | AFR | $560 | 84,100,000 | 0.81 | 16 | $1.08 ($0.33–2.72) |
| Costa Rica | UMIC | AMR | $12,030 | 5,000,000 | 0.94 | 14 | $6.67 ($2.52–15.13) |
| Côte d'Ivoire | LMIC | AFR | $1,720 | 25,100,000 | 0.82 | 19 | $1.91 ($0.66–4.53) |
| Cuba | UMIC | AMR | $8,100 | 11,300,000 | 0.99 | 12 | $5.76 ($2.43–11.99) |
| Djibouti | LMIC | EMR | $2,050 | 959,000 | 0.84 | 16 | $4.30 ($1.65–9.42) |
| Dominica | UMIC | AMR | $7,030 | 71,600 | 0.94 | 11 | $15.51 ($4.60–40.43) |
| Dominican Republic | UMIC | AMR | $7,650 | 10,600,000 | 0.94 | 9 | $4.88 ($1.97–10.68) |
| Ecuador | UMIC | AMR | $6,340 | 17,100,000 | 0.85 | 16 | $3.26 ($1.11–7.82) |
| Egypt | LMIC | EMR | $2,550 | 98,400,000 | 0.95 | 16 | $2.39 ($0.82–5.58) |
| El Salvador | LMIC | AMR | $4,060 | 6,420,000 | 0.81 | 10 | $3.08 ($1.09–7.21) |
| Equatorial Guinea | UMIC | AFR | $10,170 | 1,310,000 | 0.25 | 16 | $1.58 ($0.09–7.91) |
| Eritrea | LIC | AFR | $730 | 3,180,000 | 0.95 | 10 | $3.69 ($1.18–9.02) |
| Ethiopia | LIC | AFR | $770 | 109,000,000 | 0.72 | 13 | $0.84 ($0.23–2.25) |
| Fiji | UMIC | WPR | $6,200 | 883,000 | 0.99 | 19 | $9.56 ($4.07–19.83) |
| Gabon | UMIC | AFR | $8,030 | 2,120,000 | 0.70 | 9 | $3.55 ($0.88–10.09) |
| Gambia | LIC | AFR | $710 | 2,280,000 | 0.93 | 10 | $3.72 ($1.14–9.22) |
| Georgia | UMIC | EUR | $4,340 | 3,730,000 | 0.93 | 11 | $5.09 ($2.20–10.70) |
| Ghana | LMIC | AFR | $2,200 | 29,800,000 | 0.97 | 5 | $3.23 ($0.94–8.56) |
| Grenada | UMIC | AMR | $10,830 | 111,000 | 0.96 | 11 | $16.64 ($5.31–41.64) |
| Guatemala | LMIC | AMR | $4,550 | 17,200,000 | 0.86 | 15 | $3.01 ($1.12–6.81) |
| Guinea | LIC | AFR | $890 | 12,400,000 | 0.45 | 7 | $0.70 ($0.08–2.67) |
| Guinea-Bissau | LIC | AFR | $780 | 1,870,000 | 0.88 | 17 | $3.29 ($1.24–7.12) |
| Guyana | UMIC | AMR | $4,630 | 779,000 | 0.95 | 14 | $7.90 ($3.41–16.32) |
| Haiti | LIC | AMR | $870 | 11,100,000 | 0.64 | 18 | $1.12 ($0.29–3.05) |
| Honduras | LMIC | AMR | $2,480 | 9,590,000 | 0.90 | 18 | $3.29 ($1.36–6.90) |
| India | LMIC | SEAR | $2,020 | 1,353,000,000 | 0.89 | 14 | $1.12 ($0.26–3.22) |
| Indonesia | LMIC | SEAR | $3,890 | 267,700,000 | 0.79 | 18 | $1.40 ($0.32–4.16) |
| Iran | UMIC | EMR | $5,270 | 81,800,000 | 0.99 | 8 | $3.33 ($1.26–7.45) |
| Iraq | UMIC | EMR | $5,880 | 38,400,000 | 0.84 | 15 | $2.61 ($0.84–6.40) |
| Jamaica | UMIC | AMR | $5,360 | 2,930,000 | 0.97 | 13 | $6.46 ($2.93–13.05) |
| Jordan | UMIC | EMR | $4,250 | 9,960,000 | 0.96 | 17 | $4.56 ($1.9–9.39) |
| Kazakhstan | UMIC | EUR | $9,330 | 18,300,000 | 0.98 | 16 | $5.39 ($1.96–12.33) |
| Kenya | LMIC | AFR | $1,710 | 51,400,000 | 0.92 | 18 | $2.24 ($0.81–5.12) |
| Kiribati | LMIC | WPR | $1,630 | 116,000 | 0.95 | 12 | $9.77 ($2.85–24.81) |
| Korea, Democratic People’s Republic of | LIC | SEAR | $1,030 | 25,500,000 | 0.97 | 7 | $2.71 ($0.85–6.84) |
| Kyrgyz Republic | LMIC | EUR | $1,280 | 6,320,000 | 0.94 | 10 | $3.45 ($1.30–7.65) |
| Lao People’s Democratic Republic | LMIC | WPR | $2,570 | 7,060,000 | 0.68 | 19 | $1.87 ($0.52–5.05) |
| Lebanon | UMIC | EMR | $8,270 | 6,850,000 | 0.83 | 18 | $4.08 ($1.31–10.27) |
| Lesotho | LMIC | AFR | $1,320 | 2,110,000 | 0.93 | 13 | $4.28 ($1.73–9.05) |
| Liberia | LIC | AFR | $670 | 4,820,000 | 0.84 | 16 | $2.26 ($0.82–5.08) |
| Libya | UMIC | EMR | $7,240 | 6,680,000 | 0.97 | 17 | $5.98 ($2.41–12.91) |
| Macedonia, North | UMIC | EUR | $6,080 | 2,080,000 | 0.91 | 14 | $6.00 ($2.53–12.80) |
| Madagascar | LIC | AFR | $460 | 26,300,000 | 0.75 | 17 | $1.09 ($0.32–2.77) |
| Malawi | LIC | AFR | $390 | 18,100,000 | 0.92 | 9 | $1.99 ($0.55–5.26) |
| Malaysia | UMIC | WPR | $11,240 | 31,500,000 | 0.99 | 15 | $5.25 ($1.79–12.44) |
| Maldives | UMIC | SEAR | $10,220 | 516,000 | 0.99 | 17 | $12.43 ($4.96–27.26) |
| Mali | LIC | AFR | $900 | 19,100,000 | 0.71 | 16 | $1.22 ($0.37–3.11) |
| Marshall Islands | UMIC | WPR | $3,620 | 58,400 | 0.81 | 16 | $8.99 ($2.52–23.21) |
| Mauritania | LMIC | AFR | $1,220 | 4,400,000 | 0.81 | 10 | $2.46 ($0.81–5.88) |
| Mauritius | UMIC | AFR | $11,240 | 1,270,000 | 0.97 | 10 | $9.68 ($3.85–21.68) |
| Mexico | UMIC | AMR | $9,700 | 126,000,000 | 0.88 | 14 | $2.71 ($0.74–7.30) |
| Micronesia | LMIC | WPR | $3,060 | 113,000 | 0.75 | 19 | $6.10 ($1.72–15.82) |
| Moldova, Republic of | LMIC | EUR | $3,190 | 3,550,000 | 0.93 | 20 | $4.83 ($2.06–9.91) |
| Mongolia | LMIC | WPR | $4,100 | 3,170,000 | 0.99 | 19 | $6.41 ($2.83–12.99) |
| Montenegro | UMIC | EUR | $8,760 | 622,000 | 0.87 | 9 | $7.88 ($2.57–19.57) |
| Morocco | LMIC | EMR | $3,240 | 36,000,000 | 0.99 | 16 | $3.55 ($1.41–7.60) |
| Mozambique | LIC | AFR | $490 | 29,500,000 | 0.80 | 15 | $1.25 ($0.40–3.06) |
| Myanmar | LMIC | SEAR | $1,330 | 53,700,000 | 0.91 | 9 | $1.97 ($0.70–4.54) |
| Namibia | UMIC | AFR | $5,930 | 2,450,000 | 0.89 | 18 | $5.48 ($2.15–11.99) |
| Nepal | LIC | SEAR | $1,030 | 28,100,000 | 0.91 | 13 | $2.12 ($0.81–4.61) |
| Nicaragua | LMIC | AMR | $2,030 | 6,470,000 | 0.98 | 8 | $4.42 ($1.62–10.12) |
| Niger | LIC | AFR | $410 | 22,400,000 | 0.79 | 16 | $1.24 ($0.38–3.08) |
| Nigeria | LMIC | AFR | $2,030 | 196,000,000 | 0.57 | 18 | $0.67 ($0.11–2.34) |
| Pakistan | LMIC | EMR | $1,470 | 212,000,000 | 0.75 | 16 | $0.96 ($0.25–2.70) |
| Palau | UMIC | WPR | $17,320 | 17,900 | 0.95 | 14 | $29.11 ($7.31–82.56) |
| Panama | UMIC | AMR | $15,580 | 4,180,000 | 0.88 | 17 | $6.39 ($1.99–16.34) |
| Papua New Guinea | LMIC | WPR | $2,720 | 8,610,000 | 0.61 | 13 | $1.48 ($0.35–4.39) |
| Paraguay | UMIC | AMR | $5,870 | 6,960,000 | 0.88 | 17 | $4.22 ($1.6–9.47) |
| Peru | UMIC | AMR | $6,950 | 32,000,000 | 0.84 | 16 | $2.87 ($0.90–7.21) |
| Philippines | LMIC | WPR | $3,100 | 107,000,000 | 0.65 | 16 | $1.04 ($0.22–3.23) |
| Romania | UMIC | EUR | $12,300 | 19,500,000 | 0.86 | 17 | $4.06 ($1.17–10.82) |
| Russian Federation | UMIC | EUR | $11,290 | 144,000,000 | 0.97 | 17 | $3.75 ($0.95–10.18) |
| Rwanda | LIC | AFR | $770 | 12,300,000 | 0.97 | 19 | $2.91 ($1.13–6.26) |
| Samoa | LMIC | WPR | $4,390 | 196,000 | 0.34 | 18 | $2.17 ($0.19–9.41) |
| São Tomé and Principe | LMIC | AFR | $2,000 | 211,000 | 0.95 | 18 | $8.64 ($3.33–18.85) |
| Senegal | LIC | AFR | $1,520 | 15,900,000 | 0.81 | 23 | $2.01 ($0.67–4.83) |
| Serbia | UMIC | EUR | $7,230 | 6,980,000 | 0.96 | 8 | $5.63 ($2.25–12.42) |
| Sierra Leone | LIC | AFR | $520 | 7,650,000 | 0.90 | 20 | $2.34 ($0.85–5.29) |
| Solomon Islands | LMIC | WPR | $2,160 | 653,000 | 0.85 | 12 | $4.96 ($1.76–11.49) |
| Somalia | LIC | EMR | $500 | 15,000,000 | 0.42 | 10 | $0.52 ($0.07–1.96) |
| South Africa | UMIC | AFR | $6,340 | 57,800,000 | 0.74 | 18 | $1.90 ($0.44–5.54) |
| South Sudan | LIC | AFR | $780 | 11,000,000 | 0.49 | 15 | $0.73 ($0.13–2.46) |
| Sri Lanka | LMIC | SEAR | $4,100 | 21,700,000 | 0.99 | 20 | $4.31 ($1.60–9.52) |
| St. Lucia | UMIC | AMR | $10,320 | 182,000 | 0.95 | 21 | $14.16 ($4.95–33.15) |
| St. Vincent and the Grenadines | UMIC | AMR | $7,380 | 110,000 | 0.97 | 24 | $15.48 ($5.48–35.96) |
| Sudan | LMIC | EMR | $980 | 41,800,000 | 0.93 | 19 | $2.10 ($0.75–4.82) |
| Suriname | UMIC | AMR | $5,950 | 576,000 | 0.95 | 18 | $9.13 ($3.83–19.41) |
| Swaziland | LMIC | AFR | $4,140 | 1,140,000 | 0.90 | 20 | $6.07 ($2.49–12.86) |
| Syrian Arab Republic | LMIC | EMR | $1,860 | 16,900,000 | 0.47 | 18 | $0.83 ($0.13–2.94) |
| Tajikistan | LMIC | EUR | $830 | 9,100,000 | 0.96 | 20 | $3.06 ($1.20–6.52) |
| Tanzania, United Republic of | LIC | AFR | $1,050 | 56,300,000 | 0.98 | 18 | $2.36 ($0.85–5.38) |
| Thailand | UMIC | SEAR | $7,270 | 69,400,000 | 0.97 | 20 | $3.84 ($1.09–9.89) |
| Timor-Leste | LMIC | SEAR | $2,040 | 1,270,000 | 0.83 | 20 | $3.93 ($1.53–8.56) |
| Togo | LIC | AFR | $670 | 7,890,000 | 0.88 | 18 | $2.30 ($0.86–5.08) |
| Tonga | LMIC | WPR | $4,360 | 103,000 | 0.81 | 24 | $8.26 ($2.57–20.26) |
| Tunisia | LMIC | EMR | $3,450 | 11,600,000 | 0.97 | 19 | $4.35 ($1.78–9.12) |
| Turkey | UMIC | EUR | $9,310 | 82,300,000 | 0.98 | 21 | $4.19 ($1.07–11.32) |
| Turkmenistan | UMIC | EUR | $6,970 | 5,850,000 | 0.99 | 20 | $6.59 ($2.54–14.41) |
| Tuvalu | UMIC | WPR | $3,700 | 11,500 | 0.89 | 21 | $17.30 ($4.50–46.31) |
| Uganda | LIC | AFR | $640 | 42,700,000 | 0.93 | 21 | $1.90 ($0.63–4.50) |
| Ukraine | LMIC | EUR | $3,100 | 44,600,000 | 0.50 | 18 | $0.86 ($0.13–3.07) |
| Uzbekistan | LMIC | EUR | $1,530 | 33,000,000 | 0.98 | 19 | $2.91 ($1.11–6.40) |
| Vanuatu | LMIC | WPR | $3,030 | 293,000 | 0.85 | 20 | $6.52 ($2.40–14.52) |
| Venezuela | UMIC | AMR | $14,200 | 28,900,000 | 0.60 | 23 | $2.07 ($0.26–7.86) |
| Vietnam | LMIC | WPR | $2,560 | 95,500,000 | 0.75 | 15 | $1.31 ($0.37–3.51) |
| Yemen | LMIC | EMR | $940 | 28,500,000 | 0.65 | 18 | $0.97 ($0.25–2.71) |
| Zambia | LMIC | AFR | $1,540 | 17,400,000 | 0.90 | 18 | $2.55 ($1.00–5.49) |
| Zimbabwe | LIC | AFR | $2,150 | 14,400,000 | 0.89 | 19 | $2.82 ($1.11–6.12) |

^a^ LIC: Gross national income (GNI) per capita of $1,025 or less; LMIC: GNI per capita of $1,026 to $3,995; UMIC: GNI per capita of $3,996 to $12,375 (World Bank 2019).

^b^ Routine childhood immunization schedule includes vaccines for children aged 0-59 months.

Note: AFR = African region; AMR = Region of the Americas; DTP3 = diphtheria-tetanus-pertussis third dose coverage; EMR = Eastern Mediterranean region; EUR = European region; GDP = gross domestic product; LIC = low-income; LMIC = lower middle-income; SEAR = Southeast Asian region; UMIC = upper middle-income; WPR = Western Pacific region.

**Appendix Table C. Predicted economic cost per dose in 2018 for routine childhood vaccine delivery by cost category and world region/income level: median and interquartile range (unweighted).**

| **Global** | **Total cost per dose** | **Labor cost per dose** | **Supply chain cost per dose** | **Service delivery cost per dose** | **Capital cost per dose** |
| --- | --- | --- | --- | --- | --- |
| Global | $3.78 ($2.91–4.90) | $1.49 ($1.15–1.94) | $0.52 ($0.39–0.69) | $1.29 ($0.96–1.73) | $0.43 ($0.32–0.58) |
| **Region** | **Total cost per dose** | **Labor cost per dose** | **Supply chain cost per dose** | **Service delivery cost per dose** | **Capital cost per dose** |
| Africa | $2.44 ($1.88–3.19) | $0.97 ($0.74–1.26) | $0.34 ($0.25–0.45) | $0.83 ($0.62–1.12) | $0.28 ($0.20–0.38) |
| Americas | $5.28 ($4.04–6.95) | $2.08 ($1.59–2.76) | $0.73 ($0.54–0.98) | $1.80 ($1.32–2.44) | $0.60 ($0.44–0.82) |
| Eastern Mediterranean | $2.35 ($1.79–3.14) | $0.93 ($0.71–1.23) | $0.33 ($0.24–0.45) | $0.81 ($0.59–1.09) | $0.27 ($0.19–0.37) |
| Europe | $4.07 ($3.14–5.29) | $1.61 ($1.24–2.09) | $0.56 ($0.42–0.75) | $1.38 ($1.02–1.87) | $0.46 ($0.34–0.62) |
| Southeast Asia | $3.47 ($2.70–4.48) | $1.38 ($1.07–1.78) | $0.48 ($0.36–0.64) | $1.19 ($0.89–1.57) | $0.39 ($0.29–0.53) |
| Western Pacific | $5.76 ($4.26–7.85) | $2.27 ($1.68–3.14) | $0.80 ($0.58–1.11) | $1.96 ($1.41–2.77) | $0.66 ($0.47–0.92) |
| **Income level** | **Total cost per dose** | **Labor cost per dose** | **Supply chain cost per dose** | **Service delivery cost per dose** | **Capital cost per dose** |
| Low-income | $1.60 ($1.19–2.19) | $0.64 ($0.47–0.86) | $0.22 ($0.16–0.31) | $0.55 ($0.40–0.76) | $0.18 ($0.13–0.26) |
| Lower middle-income | $3.05 ($2.36–3.97) | $1.21 ($0.93–1.57) | $0.42 ($0.31–0.56) | $1.04 ($0.78–1.39) | $0.35 ($0.26–0.47) |
| Upper middle-income | $4.92 ($3.16–7.08) | $2.20 ($1.68–2.92) | $0.77 ($0.57–1.04) | $1.90 ($1.39–2.59) | $0.63 ($0.47–0.86) |

Note: Point estimates present the median by category; parentheses include the 25^th^ and 75^th^ percentile estimates by category. Countries included in each World Health Organization (WHO) region are low- and middle-income countries according to World Bank income level in 2019 [12].

**Appendix Table D. Sensitivity analysis for regressions of routine childhood delivery unit cost per dose on predictors: excluding two outliers with cost-per-dose estimates below $0.01.**

| Variable | Mean coefficient |
| --- | --- |
| Labor intercept | 0.12 (0.23) |
| Supply chain intercept | -0.92 (0.26) |
| Service delivery intercept | -0.02 (0.29) |
| Capital intercept | -0.99 (0.32) |
| Year | -0.10 (0.13) |
| Economic cost indicator | -0.06 (0.14) |
| Single antigen indicator | -0.57 (0.22) |
| log(doses) | 0.02 (0.12) |
| log(GDP per capita) | 0.18 (0.13) |
| log(population) | -0.28 (0.12) |
| DTP3 coverage | 0.29 (0.11) |
| Alpha | 1.14 (0.10) |

Note: Continuous predictors were standardized to mean zero and unit standard deviation; thus, fitted coefficients for continuous variables (e.g., log(doses)) represent the increase in log cost per dose observed for a 1.0 standard deviation increase in the variable. Values in parentheses represent standard errors. DTP3 = diphtheria-tetanus-pertussis third dose coverage; GDP = gross domestic product.

**Appendix Table E. Predicted programmatic, economic cost per dose in 2018 for routine childhood vaccine delivery by world region: excluding two outliers with cost-per-dose estimates below $0.01.**

| **Region** | **Mean predicted cost per dose** |
| --- | --- |
| Africa | $1.75 ($0.66–4.05) |
| Americas | $2.98 ($0.99–7.34) |
| Eastern Mediterranean | $2.17 ($0.81–4.91) |
| Europe | $4.00 ($1.37–9.50) |
| Southeast Asia | $1.62 ($0.47–4.30) |
| Western Pacific | $2.36 ($0.66–6.25) |

Note: Countries included in each World Health Organization (WHO) region are low- and middle-income countries according to World Bank income level [11].

**Appendix Table F. Sensitivity analysis for regressions of routine childhood delivery unit cost per dose on predictors: weakly informative priors used for regression coefficients and variance terms.**

| Variable | Mean coefficient |
| --- | --- |
| Labor intercept | 0.03 (0.25) |
| Supply chain intercept | -1.08 (0.29) |
| Service delivery intercept | -0.13 (0.30) |
| Capital intercept | -1.32 (0.34) |
| Year | -0.16 (0.14) |
| Economic cost indicator | -0.01 (0.15) |
| Single antigen indicator | -0.45 (0.24) |
| log(doses) | 0.01 (0.12) |
| log(GDP per capita) | 0.20 (0.13) |
| log(population) | -0.31 (0.12) |
| DTP3 coverage | 0.30 (0.11) |
| Alpha | 1.08 (0.07) |

Note: Continuous predictors were standardized to mean zero and unit standard deviation; thus, fitted coefficients for continuous variables (e.g., log(doses)) represent the increase in log cost per dose observed for a 1.0 standard deviation increase in the variable. Values in parentheses represent standard errors. DTP3 = diphtheria-tetanus-pertussis third dose coverage; GDP = gross domestic product

**Appendix Table G. Sensitivity analysis for regressions of routine childhood delivery unit cost per dose on predictors: non-informative priors used for regression coefficients and variance terms.**

| Variable | Mean coefficient |
| --- | --- |
| Labor intercept | 0.02 (0.25) |
| Supply chain intercept | -1.08 (0.29) |
| Service delivery intercept | -0.13 (0.32) |
| Capital intercept | -1.32 (0.34) |
| Year | -0.16 (0.14) |
| Economic cost indicator | -0.02 (0.16) |
| Single antigen indicator | -0.45 (0.25) |
| log(doses) | 0.00 (0.13) |
| log(GDP per capita) | 0.20 (0.13) |
| log(population) | -0.31 (0.12) |
| DTP3 coverage | 0.30 (0.12) |
| Alpha | 1.08 (0.06) |

Note: Continuous predictors were standardized to mean zero and unit standard deviation; thus, fitted coefficients for continuous variables (e.g., log(doses)) represent the increase in log cost per dose observed for a 1.0 standard deviation increase in the variable. Values in parentheses represent standard errors. DTP3 = diphtheria-tetanus-pertussis third dose coverage; GDP = gross domestic product.
